# Did Solid Surfaces Enable the Origin of Life?

**DOI:** 10.3390/life11080795

**Published:** 2021-08-05

**Authors:** İrep Gözen

**Affiliations:** 1 Centre for Molecular Medicine Norway, Faculty of Medicine, University of Oslo, 0318 Oslo, Norway; irep@uio.no; 2 Department of Chemistry, Faculty of Mathematics and Natural Sciences, University of Oslo, 0315 Oslo, Norway

**Keywords:** protocell, compartment, solid interface, lipid, origin of life

## Abstract

In this perspective article, I discuss whether and how solid surfaces could have played a key role in the formation of membranous primitive cells on the early Earth. I argue why surface energy could have been used by prebiotic amphiphile assemblies for unique morphological transformations, and present recent experimental findings showing the surface-dependent formation and behavior of sophisticated lipid membrane structures. Finally, I discuss the possible unique contributions of such surface-adhered architectures to the transition from prebiotic matter to living systems.

## 1. Introduction

Primitive cell formation and development is closely linked to the origin of life, which is considered to be one of the unsolved fundamental scientific problems. Researchers seek answers in the laboratory by simulating early Earth conditions as they existed approximately 3.8 billion years ago, with the aim to instantiate autonomously forming primitive compartments that can develop into self-sustaining and reproducing cells. 

Different types of model structures are used to mimic protocells, the hypothetical precursors of living cells. One category is based on droplets, formed via liquid–liquid phase separation [1,2]. A prominent example is coacervates [3,4], droplets concentrated with macromolecules, dispersed in a more dilute liquid phase. The other category of protocell models comprises amphiphiles, such as fatty acids or phospholipids, in aqueous environments [3,5,6]. Amphiphiles can spontaneously self-assemble to form spherical membrane compartments freely suspended in an aqueous phase. Lipid membranes envelope modern cells, and are ubiquitous among almost all cellular structures today. It has been shown that lipids could have been synthesized under prebiotic conditions [7,8] or delivered to the early Earth by meteorites [9,10]. The potential availability of lipid species at that time, and their ability to spontaneously self-assemble to ordered membranous structures, are two arguments in favor of liposomal compartments as plausible building blocks of primitive cells. Naturally present were, besides water, rocks and minerals as fundamental constituents of the early Earth. The possible role of solid surfaces in the process of primitive cell formation and development might have been largely overlooked.

In this perspective article, I discuss the possible involvement of solid surfaces in the emergence of membranous primitive cells on the early Earth. I briefly explain why surfaces intrinsically possess energy, and how it can be used by biosurfactants for morphological transformations. I then progress to recent experimental observations of solid surface-dependent membrane transformation pathways that consistently lead to robust model protocells. Finally, I discuss the variety of resulting unique primitive structures, their potential advantages, and possible contributions to the transition from non-living to animated matter.

## 2. Intrinsic Energy of Surfaces

**Figure 1:**
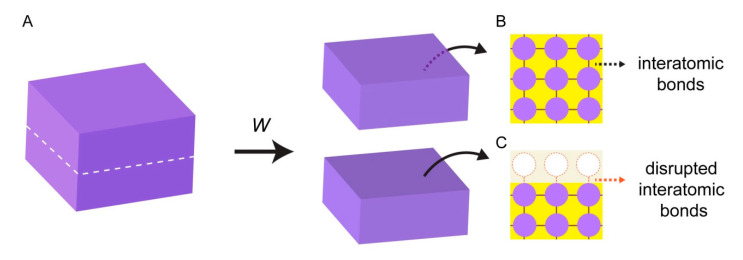
Surface energy. (**A**) Block of solid material. Work (*W*) is performed to split the solid block into two pieces. Inside the bulk phase of the block, the atoms experience attractive forces established with all their neighboring atoms (**B**). Atoms located at the newly created interface have disrupted, unbalanced cohesive interactions (**C**).

Surface energy emerges from the interactions of individual atoms at interfaces. When a block of a solid material is cut into two pieces (Figure 1A), two new solid interfaces are being created. In the bulk (unaltered) phase of the material, atoms maintain bonds with all surrounding atoms, and experience attractive interactions in all directions (Figure 1B). Interatomic bonds at the broken interface, however, are now disrupted, and the surface atoms are lacking favorable interactions (Figure 1C).

Work (*W*) needs to be performed to break the bonds between the atoms, and to create new surfaces. The work that is performed to create each unit area of a new surface is equivalent of surface energy or surface tension (*σ*) [11,12]. The magnitude of the work is associated with the nature of the bonds in the material. High energy surfaces (*σ* > 200 mN/m [12,13]) are composed of atoms with strong bonds such as covalent, ionic, or metallic bonds. Most natural surfaces including metals, diamond, silica glasses, and ceramics fall under this category. The atoms at low energy surfaces (*σ* < 50 mN/m [12,13].) e.g., plastics or resins, are attached to each other with rather weak bonds, e.g., van der Waals or hydrogen bonds.

All high energy surfaces will tend to reduce their surface energy, hence the overall Gibbs free energy. If surfaces cannot establish bonds with atoms of their own kind, they will seek physical contact with other matter, e.g., water or surface-active molecules (surfactants), and reduce their surface energy. The energy of a solid surface can therefore be harnessed by other soft and deformable materials with the ability to cover the surface. In this context, the interaction of biological and biomimetic membranes, consisting largely of lipids, with solid high energy surfaces is of special interest for the autonomous formation of primitive protocells [14].

Solid surfaces were abundant on the early Earth in the form of minerals and rocks, which are defined as aggregates of several minerals. From the beginning of planet formation until today, there have been 10 stages of mineral evolution, and each of them increased mineral diversity on the Earth [15,16]. Stages 1–6 occurred during Accretion, the Hadean eon and the following Eoarchean era, all together the ‘early Earth’ (4.6–3.6 Gya). During this period, about 1500 different mineral species appeared [15,16]. Several minerals have been shown to be able to catalyze peptide, lipid, and nucleic acid synthesis [17]. Two specific minerals which have been described as potentially important in the context of the origin of life, are clay [18,19] and quartz (SiO_2_) [16,20]. Natural quartz specimens, along with synthetic SiO_2_ surfaces, have been used as solid substrates in the key studies that constitute the experimental foundation of my perspective.

## 3. Biomembrane Transformations on Solid Surfaces

Giant unilamellar vesicles, encapsulating an aqueous volume within a spherical lipid bilayer, are a common experimental model system for protocell studies [21]. Although much more complex both in structure and function, contemporary biological cells are also enveloped in a lipid-based membrane, and are of similar size. The potential availability of lipid species in the prebiotic environment, and their ability to spontaneously self-assemble to cell-like compartments, make lipid vesicles plausible biomimetic architectures for studies of primitive cells. Among different lipid species, the structurally simpler fatty acids are considered to have been more prevalent on the early Earth. Experimental evidence for prebiotic pathways to simple amphiphiles is well-established [22]. Monocarboxylic acids were also found in extraterrestrial sources, e.g., carbonaceous meteorites [9,10]. However, experimental findings suggest that phospholipids could have also been present under prebiotic conditions [7,8,23].

**Figure 2:**
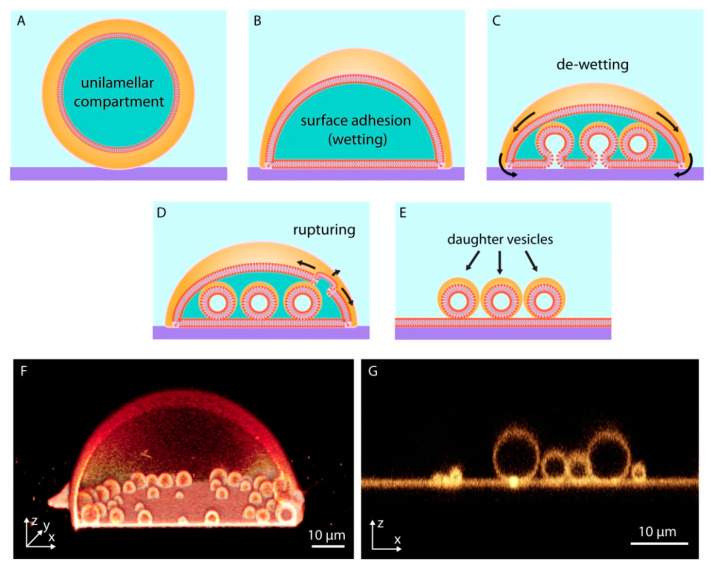
Surface-enhanced subcompartmentalization and pseudo-division of model protocells. An isolated giant unilamellar compartment (**A**), adheres on a solid substrate upon contact (**B**). Reversed adhesion leads to the de-wetting of the substrate, release of small membrane regions from the surface, and formation of small subcompartments (**C**). The disintegration of the upper (with respect to the surface) protocell membrane due to an increase in tension (**D**), leads to the transformation of daughter cells to independent surface-adhered compartments (**E**–**G**) shows confocal micrographs corresponding to (**D**,**E**).

When a giant unilamellar lipid compartment, a spherical continuous bilayer membrane, is brought in contact with a high energy solid substrate in an aqueous medium (Figure 2A), the vesicular membrane initially partially adheres onto the substrate, adopting a dome-like shape (Figure 2B). This process, in which a fluid material makes and maintains physical contact with a solid substrate, is termed ‘wetting’. Wetting of surfaces by lipid membranes, which display two-dimensional fluid properties, occurs in various forms [24] depending on the surface material, the overall surface energy, and the composition of the lipid membrane.

The membrane-substrate adhesion can be enhanced by the presence of multivalent ions, e.g., Ca^2+^, Mg^2+^, in the aqueous environment surrounding the compartment. If the adhesion weakens, for example due to a decrease in the concentration of the ions in the surrounding solution, the membrane starts to partially de-wet and lift off from the surface (Figure 2C). The released membrane regions and newly formed invaginations possess nanoscale membrane curvature. Membrane tension rises because at these highly curved regions, the distance between the individual lipid molecules increases and their hydrophobic moieties become exposed to water [25]. The locally high tension initiates flow of lipid material from membrane areas of lower tension towards high tension regions (Marangoni flow) [26,27] (Figure 2C). This newly arriving material transforms the membrane invaginations over time into giant spherical compartments. These membranous subcompartments show resemblance to membrane-enclosed organelles inside modern cells; they were shown to take up, and concentrate ambient compounds, similar to cellular organelles [27].

The enveloping membrane can rupture and completely disintegrate, leaving behind the subcompartments (Figure 2D), each of which can thus be considered an individual daughter protocell (Figure 2E). This phenomenon may well be viewed as a pseudo-division mechanism of primitive cells, which were certainly lacking the complex biological machinery required to enable contraction and fission. Figure 2F shows a 3D cross-sectional confocal micrograph of a subcompartmentalized protocell and Figure 2G the daughter cells after disintegration of the enveloping membrane.

**Figure 3:**
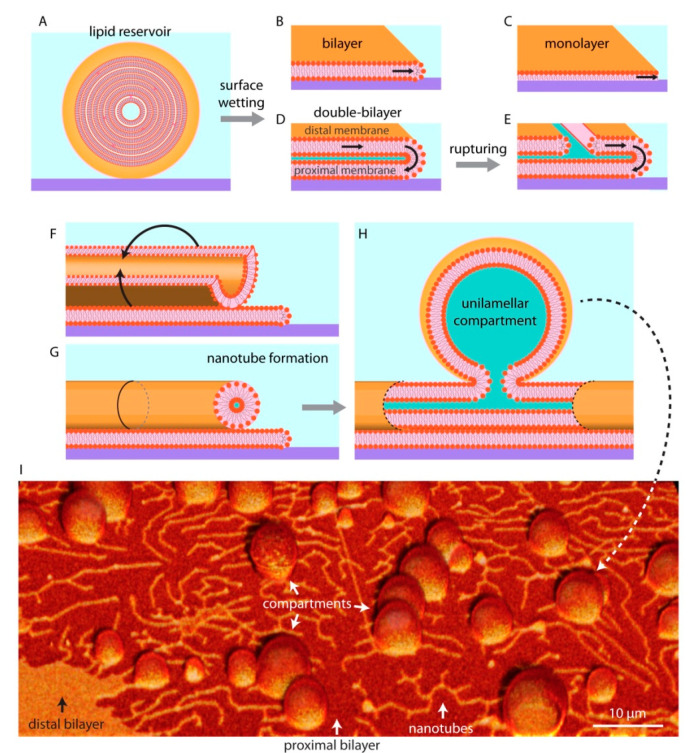
Lipid reservoir-surface interactions. When multilamellar vesicles, i.e., lipid reservoirs, (**A**) come in physical contact with solid surfaces, they can wet the surface in different ways and can spread as a bilayer (**B**), monolayer (**C**), or a double bilayer (**D**). The distal membrane (upper bilayer of a double bilayer membrane with respect to the surface) ruptures due to an increase in membrane tension (**E**). The distal membrane regions remaining on the proximal (lower) membrane wrap up (**F**) to form lipid nanotubes (**G**), alleviating tension at the membrane edges. Fragments of nanotubes swell over time (**H**), resulting in protocell-nanotube networks. (**I**) Confocal micrograph showing protocells connected via lipid nanotubes. Please note that the schematic drawings are not to scale: the lipid nanotubes are typically 100 nm in diameter, where a lipid bilayer is about 5 nm thick.

Unilamellar compartments are not the only known kind of self-assembled lipid structures. If there is sufficient lipid material available, they can form multilamellar vesicles (MLV), in which several lipid bilayers are packed on top of each other in an onion shell-like fashion (Figure 3A). The individual layers in such structure are interconnected through membrane defects, and can thus be viewed as a single large fluid reservoir. When a MLV is brought in contact with a high energy substrate in an aqueous surrounding (Figure 3A), it spreads isotropically and wets the entire available surface until the accessible lipid material in the reservoir is completely depleted. On glass, the membranes generally spread as a bilayer [28,29] (Figure 3B), while on hydrophobic surfaces as a monolayer [11,12,30] (Figure 3C). The most commonly experimentally investigated form of bio (mimetic) membranes is the bilayer, as the plasma membrane of contemporary cells as well as most of the intracellular membranes are single lipid bilayers.

On SiO_2_ and some metal oxides, the lipid reservoirs spread as a double bilayer membrane [26,31,32] (Figure 3D), where the proximal bilayer (in closer proximity to the surface) is immobilized on the solid substrate as it spreads. The distal (of greater distance to the surface) membrane is simultaneously expanding along the spreading edge in a tank thread-like motion (Figure 3D). The spreading continues until the membrane tension exceeds lysis tension (5–10 mN/m), resulting in rupturing of the distal membrane (Figure 3D) [32,33]. Some of the distal membrane areas however, especially the regions pinned to the proximal bilayer, remain intact. The curved edge of these distal membrane regions grows as the ruptures propagate, leading to an increase in membrane edge (line) energy [34]. To avoid the increasing edge energy cost, these distal membrane regions rapidly wrap into lipid nanotubes (Figure 3F,G). Lipid nanotubes are highly curved cylindrical membrane structures. They represent a local energy minimum, yet remain costly to the overall system due to high bending energy. To reduce bending energy, fragments of the nanotubes swell into spherical vesicular compartments over time (Figure 3H). A protocell network emerges in this process, consisting of several lipid compartments interconnected by lipid nanotubes [11,12,33] (Figure 3I).

The protocell networks can be formed from membranes consisting of phospholipids only, or from mixtures of phospholipids and fatty acids [35]. The networks have been shown to encapsulate water soluble compounds, even RNA and DNA [35,36,37]. Especially RNA has been a molecule of interest in origin of life studies, as it can act uniquely both as a genetic information carrier and an enzyme analog, catalyzing its own replication [38]. In an environment where molecules such as proteins were not yet available, the capability of RNA to replicate without the need for an enzyme would be an advantageous feature. The ability of model protocells to encapsulate and maintain RNA has therefore been widely investigated. The surface-adhered compartments can grow rapidly and fuse with merging of contents [36], passively replicate by transporting material through the nanotubes [39], divide, migrate, and re-attach to a surface in remote locations [33]. The protocell networks can also grow in groups in a colony-like manner, where the membranes of the individual compartments are in physical contact with each other [35,37].

## 4. Possible Implications of Self-Forming Surface-Based Protocells for the Origin of Life

Although the autonomous formation and development of unique protocell structures is not the direct equivalent of the origin of life, a strong relation has been established between self-forming and -developing protocells, and abiogenesis. For example, according to Ganti’s chemoton model [40], one of the three essential components of an elementary unit to be considered alive is a bilayer-enclosed compartment. The others are metabolism and self-replication. Nutrients are adsorbed through the membrane and incorporated into the metabolic cycle, where the waste products are released through the membrane again. Key molecular constituents of the membrane are produced by metabolic reactions as well as the “replicator unit”.

**Figure 4:**
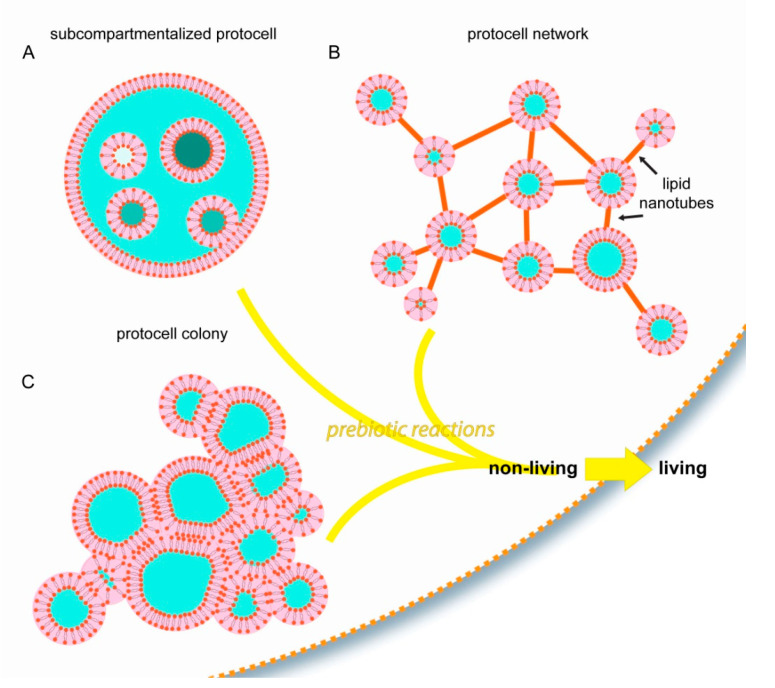
Schematic drawings summarizing the experimentally observed protocell model structures which autonomously and consistently form due to surface interactions. (**A**) Subcompartmentalized protocells with organelle-like membranous subunits, (**B**) Protocell-nanotube networks, (**C**) Protocell colonies. The possible contribution of such unique structures to the transition from non-living to living, compared to the ‘bulk hypothesis’ in which a spherical compartment freely suspended in water is assumed as first step towards life, is discussed in this article.

Most of the current studies on membranous compartmentalization focus on bulk lipid vesicle suspensions as the initial step. The examples discussed in this article comprise alternative structural starting points, all of which are based on surface pathways (Figure 4).

With the involvement of surface-membrane interactions, membranous protocellular subcompartments can rapidly, autonomously, and consistently form inside a protocell [27] (Figure 2 and Figure 4A). The subcompartments are able to encapsulate, segregate and maintain ambient compounds at different concentrations in isolated environments [27], similar to their biological counterparts in modern cells: the organelles. Until the last decade, membrane-enclosed organelles were associated exclusively with eukaryotic cells. Recent evidence indicates that membranous subunits with specific functions also exist in Prokaryota [41], e.g., bacteria [42] and archaea [43]. These new reports, and the recent findings showing the ability of surface-adhered model protocells to easily form subcompartments, support the possibility that primitive protocells, leading to the last common universal ancestor, already had separate structural subunits to support different prebiotic reactions. Furthermore, as described above (Figure 2D–G), after a protocell disintegrates, subunits may become independent daughter cells [27]. It could be that one or some of the subcompartments inside an original protocell preserved a particular chemical reaction, which provided an evolutionary advantage. An enveloping protocellular membrane would impede the impact of detrimental environmental factors on the subcompartments, which later become the daughter cells. Such system could persist, despite an often unpredictable and changing environment. 

Another type of biomembrane morphology occurring as a result of surface interactions is the interconnected micro-container network [35,36] (Figure 3 and Figure 4B). The containers in such physical network may have accommodated different prebiotic chemical reaction networks [44]. Chemical reaction networks comprise multiple chemical reactions which are connected to each other through chemical compounds that participate both as reactants and products. The nanotube protocell networks have the necessary features to serve as physical reactors for prebiotic chemical reaction networks. In this setting, it is possible that the product of one reaction is transferred to a nearby reactor via the tunneling lipid nanotubes, where it becomes a reactant in the new reaction node, in a new compartment. The protocell-nanotube networks allow the continuous or discontinuous versions of chemical reactions in networks [44], as well as both the ‘genetics first’ or ‘metabolism first’ models of the origin of life problem. Discontinuous chemical reactions follow a specific order of added reagents, or require specific steps in which chemical compounds are segregated, concentrated, purified or eliminated leading to conditions permitting the subsequent reactions [44].

The third interesting form of assembly of primitive membranes on solid substrates is the protocell colony (Figure 4C). A key feature of biological colonies is their ability to adopt properties and capabilities greater than the individual units, for example to cope with environmental conditions unsuitable for survival as individuals. It is plausible that prebiotic protocell colonies, if they existed, exhibited increased mechanical stability, and had the ability to perform complex chemical interactions and exchange information. Vesicle colonies were hypothesized earlier as possible prebiotic compartments [5], and have been shown experimentally [45,46]. However, the mechanisms of formation involve fusogenic materials that were most likely not present on the early Earth, e.g., poly(arginine) [45], or colony formation requires directed assembly [46]. Vesicle agglomerates were previously reported to assemble from lipid monomers in the presence of mineral micro- and nanoparticles [18,47,48]. New research then showed the ability of protocell colonies to spontaneously form on synthetic surfaces [35], on Hadean Earth minerals, and on a rare Martian meteorite specimen [37]. In these new reports, protocell colonies were derived from the same lipid reservoir and consisted of a membrane of identical composition.

## 5. Conclusions

The necessary assumptions for the surface-mediated processes described above are minimal: the presence of lipid assemblies in an aqueous environment, and the presence of solid surfaces, all of which are thought to have existed on the early Earth.

Solid surfaces naturally possess energy which can be used by soft materials to perform astonishing shape transformations. Both synthetic and natural forms of solid surfaces have been recently experimentally explored in this context under laboratory conditions, which led to the discovery of unique lipid assemblies: subcompartmentalized protocells, protocell-nanotube networks, and protocell colonies. Considering the necessary minimal assumptions, it is well plausible that the completely autonomous transformations described above could have occurred on rock and mineral surfaces on the early Earth, or even in similar environments on other planets. Solid natural surfaces can possibly be the key enabling factor for the emergence of primitive cells. The morphologies generated by the recently identified surface-mediated pathways are fit to be combined with prebiotic chemical reactions. As a first step towards that goal, exploration of chemical reactions involving lipids and other amphiphiles might be a suitable route, extending the ‘Lipid World hypothesis’ [49,50]. Dry-wet cycles, or fusion of new multilamellar reservoirs to existing structures, can likely provide further advancement of our understanding of the relevance of these special prebiotic morphologies for the origin of life.

I have shared here my perspective on the role of solid surfaces as potential key components for the formation of non-trivial protocell structures, networks, and colonies. Since protocells are considered a stepping stone towards life’s origin, I conclude that protocell morphologies enabled by solid surfaces might have been relevant and advantageous. I note that the emergence of life can hardly be understood from a single, isolated point of view, and I strongly advocate a transdisciplinary approach to eventually generate a coherent theory.
